# Geniposide promotes beta-cell regeneration and survival through regulating *β*-catenin/TCF7L2 pathway

**DOI:** 10.1038/cddis.2015.107

**Published:** 2015-05-07

**Authors:** D D Yao, L Yang, Y Wang, C Liu, Y J Wei, X B Jia, W Yin, L Shu

**Affiliations:** 1 Key Laboratory of New Drug Delivery System of Chinese Materia Medica, Jiangsu Province Academy of Chinese Medicine, Nanjing, China; 2 Jiangsu Key Laboratory of Xenotransplantation, Nanjing Medical University, Nanjing, China; 3 Department of Endocrinology, Jiangsu Province Hospital on Integration of Chinese and Western Medicine, Nanjing University of Chinese Medicine, Nanjing, China; 4 State Key Laboratory of Pharmaceutical Biotechnology, School of Life Sciences, Nanjing University, Nanjing, China

## Abstract

T-cell factor 7-like 2 (TCF7L2) is an important transcription factor of Wnt/*β*-catenin signaling, which has critical roles in *β*-cell survival and regeneration. In preliminary screening assay, we found geniposide, a naturally occurring compound, was able to increase TCF7L2 mRNA level in Min6 cells. Here we aimed to investigate the role of geniposide in *β*-cell and underlying mechanism involved. Geniposide was found to promote *β*-cell survival by increasing *β*-cell proliferation and decreasing *β*-cell apoptosis in cultured mouse islets after challenge with diabetic stimuli. Geniposide protected *β*-cell through activating Wnt signaling, enhanced expressions of TCF7L2 and GLP-1R, activated AKT, inhibited GSK3*β* activity, and promoted *β*-catenin nuclear translocation. The protective effect of geniposide was remarkably suppressed by siRNAs against *β*-catenin, or by ICG001 (*β*-catenin/TCF-mediated transcription inhibitor). Moreover, geniposide promoted *β*-cell regeneration *in vivo* to normalize blood glucose in high-fat diet and db/db mice. Increased *β*-cell proliferation was observed in pancreatic sections of geniposide-treated diabetic mice. Most importantly, geniposide triggered small islet-like cell clusters formation as a result of *β*-cell neogenesis from ductal epithelium, which was well correlated with the increase in TCF7L2 expression. In exocrine cells isolated from mouse pancreas, geniposide could induce duct cell differentiation through upregulating TCF7L2 expression and activating JAK2/STAT3 pathway. Taken together, we identified a novel role of geniposide in promoting *β*-cell survival and regeneration by mechanisms involving the activation of *β*-catenin/TCF7L2 signaling. Our finding highlights the potential value of geniposide as a possible treatment for type 2 diabetes.

Diabetes is characterized by *β*-cell loss and dysfunction.^1, 2^ One therapeutic strategy for diabetes is to prevent *β*-cell failure and to promote new *β*-cell formation. Lineage-tracing experiments have shown that new *β*-cells can arise from proliferation of preexisting *β*-cells.^3^ An alternative source of *β*-cells has been explored that facultative progenitors can be found in regenerating pancreatic ducts.^4^ Pancreatic duct cells are considered a potential source of *β*-cell regeneration.

In recent years, the role of Wnt/*β*-catenin pathway in type 2 diabetes mellitus (T2DM) has been increasing appreciated.^5, 6^ Wnt/*β*-catenin signaling is a key modulator for *β*-cell insulin secretion,^7^ *β*-cell neonatal growth, and regeneration.^8^ As a major transcription factor of Wnt signaling, T-cell factor 7-like 2 (TCF7L2) was shown to mediate its effects through glucagon-like peptide 1 receptor (GLP-1R) signals in our previous publications. TCF7L2 overexpression could enhance GLP-1R expression and activate downstream pathway AKT consequently,^9^ promote *β*-cell regeneration *in vivo*^10^ by triggering the conversion from ductal epithelial cell to *β*-cell, and protect *β*-cell from glucose and pro-inflammatory cytokine-mediated toxicity.^11^ Reports from other groups also confirmed that TCF7L2 was able to improve *β*-cell survival, function, and regeneration.^8, 12, 13, 14^ Also, TCF7L2/*β*-catenin itself promotes synthesis of GLP-1 in intestinal L-cells.^15^

Given the important role of TCF7L2 in *β*-cell survival, we conducted a small-scale natural-compound-screening assay in Min6 cells, with an aim to identify small molecules that can increase TCF7L2 mRNA level. Geniposide, a natural dietary pigment isolated from the gardenia fruits, has emerged as a promising candidate because it significantly increased TCF7L2 mRNA level in cells treated with high glucose.

Gardenia fruits have been used as a traditional herbal medicine that possess anti-inflammatory,^16^ antioxidative,^17^ and hypoglycemic properties.^18^ One study showed geniposide decreased the expression of glucose-6-phosphatase in a diabetic mouse model induced by 3-week high-fat diet (HFD) feeding combined with streptozotocin injection.^19^ Of note, it is not an appropriate animal model for T2DM study. In recent studies, geniposide was shown to stimulate insulin secretion in INS-1 cells in different glucose concentrations by activating GLP-1R.^20^ In addition, geniposide could counteract lipotoxicity-induced INS-1 cell apoptosis, through GLP-1R signaling.^21^ However, whether geniposide can modulate *β*-cell regeneration and whether the possible effect of geniposide is mediated by TCF7L2/*β*-catenin signaling remain largely unknown. To answer these questions, we initiated this study.

In the present study, we evaluated the effects of geniposide on *β*-cell survival and regeneration and investigated the underlying mechanisms as well. Isolated mouse islets and Min6 cells were used to explore effects of geniposide on *β*-cell survival *in vitro*. HFD-induced diabetic mice and db/db mice were used to investigate *β*-cell regeneration *in vivo*.

## Results

### Geniposide protected islet *β*-cells against glucose and pro-inflammatory cytokine-mediated toxicity by upregulating TCF7L2 expression

**Figure 1:**
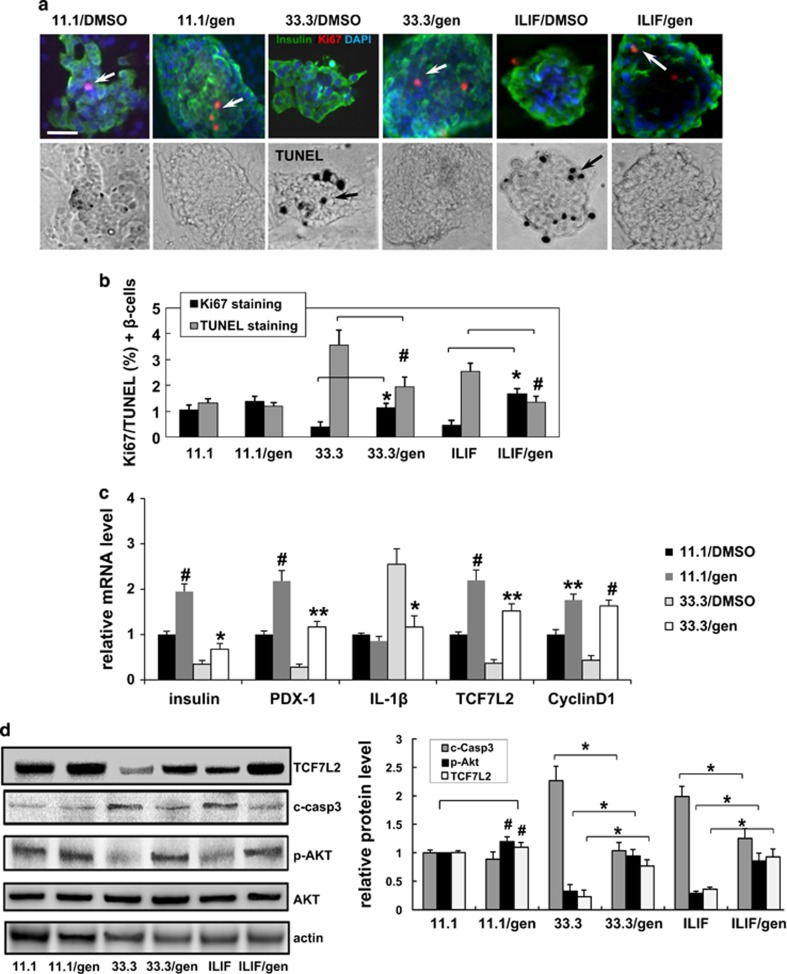
Geniposide protected islet *β*-cells by upregulating TCF7L2 expression. (**a**) Isolated mouse islets were exposed to diabetic stimuli (33.3 mM glucose or the mixture of 2 ng/ml IL-1*β* plus 1000 U/ml IFN-*γ* (ILIF)) with geniposide (20 *μ*M) or DMSO as control for 3 days. Proliferation was measured by the Ki67 staining (in red, indicated by white arrows), and apoptosis by the TUNEL assay stained in black (indicated by black arrows). Islets were triple-stained for insulin in green and counterstained for DAPI in blue. Scale bars, 20 *μ*m. (**b**) Results are expressed as means±S.E. of the percentage of Ki67-positive or TUNEL-positive *β*-cells. *P<0.05, geniposide to DMSO (Ki67 staining); ^#^*P*<0.05, geniposide to DMSO (TUNEL assay). (**c**) RT-PCR analysis of mRNA isolated from the islets cultured in 11.1 or 33.3 mM glucose with/without geniposide. Results were normalized to tubulin. Data are shown as mean±S.E. from three independent experiments. (^#^*P*<0.05, geniposide to DMSO in 11.1 mM glucose-treated islets; *P<0.01, **P<0.005, geniposide to DMSO in 33.3 mM glucose-treated islets). (**d**) Representative western blots for the diabetic stimuli-treated islets. The densitometric analyses of three independent experiments are shown. (^#^*P*<0.05, geniposide to DMSO in 11.1 mM glucose-treated islets; *P<0.05, geniposide to DMSO in 33.3 mM glucose- or ILIF-treated group)

In the present study, Ki67 immunostaining was used to examine cell proliferation, and TUNEL staining was used to assess apoptosis. The results, shown in Figures 1a and b, demonstrated that treatment with high-concentration glucose (33.3 mM) or a mixture of cytokines (IL-1*β* plus IFN-*γ* (ILIF)) reduced *β*-cell proliferation and induced *β*-cell apoptosis compared with the levels observed in 11.1 mM glucose-treated cells. Notably, islets treated with geniposide were protected against these deleterious effects. In islets treated with 33.3 mM glucose, geniposide increased *β*-cell proliferation (2.8-fold) and decreased apoptosis (1.84-fold) compared with that observed in DMSO-treated cells. Similarly, in ILIF-treated islets, geniposide increased *β*-cell proliferation 3.4-fold and decreased apoptosis up to 1.88-fold compared with that observed in DMSO-treated cells (Figure 1b). Meanwhile, the effect of geniposide on islet *β*-cell function was evaluated by GSIS (glucose-stimulated insulin secretion) assay (Supplementary Figure S3). The insulin secretion of islets was not significantly impacted by geniposide.

To confirm the preliminary result that geniposide can upregulate *TCF7L2* mRNA levels in Min6 cells, cultured mouse islets were exposed to 33.3 mM glucose as a diabetic stimulus. As shown in Figure 1c, the mRNA levels of *TCF7L2*, pancreatic and duodenal homeobox 1 (*PDX-1*), and *insulin* were remarkably reduced in islets exposed to 33.3 mM glucose compared with that in 11.1 mM glucose-treated cells. In the adult pancreas, PDX-1 is a transcription factor that regulates the expression of *β*-cell-specific genes including *insulin* and *Glut2*, also multiple aspects of *β*-cell function and survival. Here we observed that the mRNA levels of *PDX-1* and insulin were increased by geniposide 4.2-fold and 1.95-fold, respectively, compared with their levels in DMSO-treated islets. Meanwhile, *TCF7L2* mRNA levels in islets were increased 4.1-fold by geniposide treatment. *Cyclin D1*, a target gene of the *β*-catenin/TCF transcription complex, was also increased 3.7-fold by geniposide treatment than the levels in control islets.

IL-1*β* is a pro-inflammatory cytokine known to cause *β*-cell failure and destruction.^22^ A previous study showed that in human islets, glucose-induced *β*-cell apoptosis and dysfunction are partly accounted for by the IL-1*β* secreted from the *β*-cells themselves.^23^ Here we showed that exposure to 33.3 mM glucose increased IL-1*β* mRNA levels in cultured islets, and this increase was significantly suppressed by geniposide treatment (Figure 1c).

Simultaneously, TCF7L2 expression in treated islets was measured by western blotting (Figure 1d). We previously showed that exposure to high glucose or a cytokine mixture decreased TCF7L2 expression in islets.^11^ Here we showed that geniposide treatment significantly restored the impaired TCF7L2 expression in high glucose- or cytokine mixture-treated islets. Moreover, the decrease in AKT phosphorylation and the increase in caspase-3 cleavage induced by high glucose or the cytokine mixture were also largely reversed by geniposide treatment. In 11.1 mM glucose-cultured islets, TCF7L2 expression and p-AKT levels also appeared to be enhanced by geniposide treatment; however, the caspase-3 cleavage was not significantly affected.

### Geniposide activated *β*-catenin/TCF7L2 signaling to prevent glucose and pro-inflammatory cytokine-mediated toxicity

**Figure 2:**
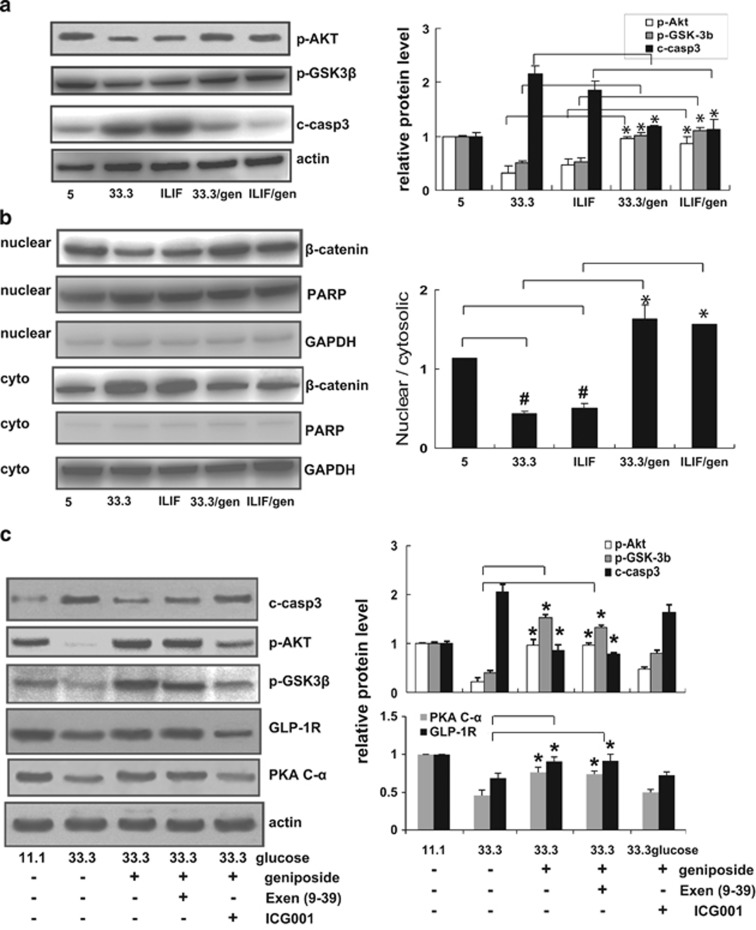
Geniposide activated *β*-catenin/TCF7L2 signaling in *β*-cell. (**a**) Representative western blots from the diabetic stimuli-treated Min6 cells. (**b**) Western blot analysis of the *β*-catenin cellular distribution in Min6 cells after treatment with geniposide. The densitometric analyses of three independent experiments are shown. (^#^*P*<0.05 to 5 mM glucose-cultured min6 cells; *P<0.05, geniposide to DMSO). (**c**) Western blot analysis of effects of Exen (9–39) (a GLP-1R antagonist; 200 nM) or ICG001 (a *β*-catenin/TCF-mediated transcription inhibitor; 25 *μ*M) on geniposide-mediated *β*-cell protection. Data are shown as mean±S.E. from three independent experiments. (*P<0.05, geniposide to DMSO)

In general, TCF7L2 exerts its biological activity through *β*-catenin/TCF7L2-mediated transcription. In this study, the effect of geniposide on *β*-catenin translocation was first examined in Min6 cells. Consistent with the results from islets, in Min6 cells, the diabetic stimuli decreased p-AKT and p-GSK3*β* levels. However, geniposide treatment increased the p-AKT and p-GSK3*β* levels, which can promote *β*-catenin stabilization and translocation (Figure 2a). The western blot analysis revealed that in Min6 cells under diabetic stimuli, *β*-catenin predominantly accumulated in the cytoplasmic fraction (Figure 2b). However, after geniposide treatment, *β*-catenin translocated into the nucleus, and the nuclear/cytosolic ratio of *β*-catenin was significantly elevated than that observed in DMSO-treated cells.

To further investigate the role of *β*-catenin/TCF7L2 and GLP-1R in geniposide's effect, islets were treated with exendin (9–39), an antagonist of GLP-1R, and ICG001, a transcriptional inhibitor of *β*-catenin/TCF. In 33.3 mM glucose-exposed cells, the regulatory effects of geniposide on p-AKT, p-GSK3*β*, and caspase-3 cleavage were significantly suppressed by ICG001, but not significantly prevented by exendin (9–39) (Figure 2c). The classical GLP-1R signaling in pancreatic *β*-cells mediates PI3K/AKT activation and PKA activation by increasing cAMP levels.^24^ In this study, expression of the PKA catalytic subunit PKA C-*α* was examined. Again, geniposide restored the decreased PKA C-*α* expression induced by 33.3 mM glucose exposure, meanwhile, this effect was strongly suppressed by ICG001, but was not affected by exendin (9–39). Downregulation of GLP-1 and GIP receptor expression in hyperglycemia have been reported in our previous studies^9^ as well as other publications.^25, 26^ Interestingly, here we observed that geniposide can upregulate GLP-1R expression, which may explain the different effects of ICG001 and exendin (9–39) on the effect of geniposide.

### Geniposide protected *β*-cell survival from glucose-mediated toxicity in a *β*-catenin/TCF7L2 signaling-dependent manner

**Figure 3:**
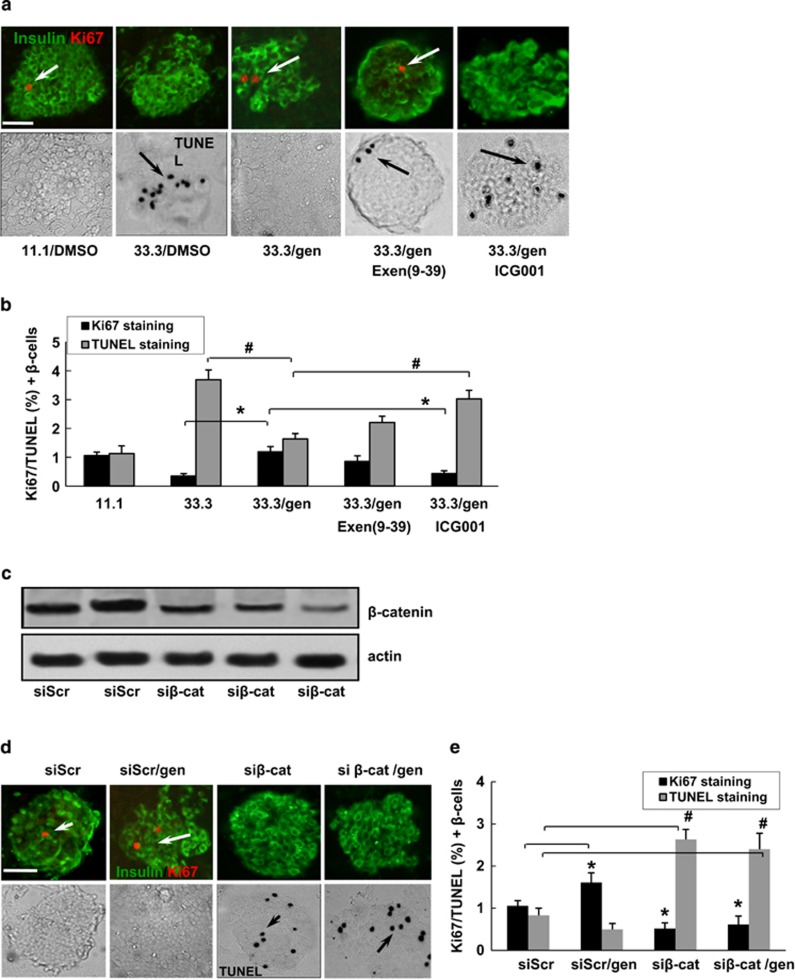
Involvement of *β*-catenin/TCF7L2 signaling in the protective effect of geniposide on islets *β*-cell survival. (**a**) Isolated mouse islets were exposed to 33.3 mM glucose with/without geniposide, Exen (9–39), or ICG001 for 3 days. The *β*-cell proliferation was measured by the Ki67 staining (in red), and apoptosis by the TUNEL assay stained in black. Islets were co-stained for insulin in green. (**b**) Results are expressed as means±S.E. of the percentage of Ki67-positive or TUNEL-positive *β*-cells. *P<0.05 (Ki67 staining); ^#^*P*<0.05 (TUNEL assay), 33.3/gen to 33.3 or 33.3/gen/ICG001 to 33.3/gen. Scale bars, 20 *μ*m. (**c**) Representative western blots from islets transfected with siScr or si*β*-cat. (**d**) Isolated mouse islets were treated by siScr or si*β*-cat with/without geniposide for 3 days. Proliferation was measured by the Ki67 staining (in red), and apoptosis by the TUNEL assay stained in black. Islets were co-stained for insulin in green. (**e**) Results are expressed as means±S.E. of the percentage of Ki67-positive or TUNEL-positive *β*-cells. *P<0.05 to DMSO (Ki67 staining); ^#^*P*<0.05 to DMSO (TUNEL assay). Scale bars, 20 *μ*m

Effects of exendin (9–39) and ICG001 on *β*-cell turnover were examined in 33.3 mM glucose-treated mouse islets by Ki67 and TUNEL staining assays (Figures 3a and b). Consistent with results from western blotting assay, the protective effect of geniposide on *β*-cell survival was suppressed by treatment with ICG001, largely attenuated by ICG001(Figure 3b, *P<0.05, Ki67 staining in 33.3/gen group *versus* 33.3/gen+ICG001 group, ^#^*P*<0.05, TUNEL staining in 33.3/gen group *versus* 33.3/gen+ICG001 group), but remained unaffected in the presence of exendin (9–39) treatment.

To further confirm the involvement of *β*-catenin/TCF7L2 in geniposide's effect, *β*-catenin expression in isolated mouse islets was reduced by siRNA-mediated gene silencing as described previously.^11^ As shown in Figure 3c, the *β*-catenin siRNA (si*β*-cat) decreased *β*-catenin expression by 65.6% compared with the levels in the scramble siRNA (siScr)-transfected islets. We transfected 100 nM si*β*-cat to deplete *β*-catenin, and then measured the effect of geniposide on *β*-cell survival by immunostaining (Figures 3d and e). In the siScr group, *β*-cell proliferation in geniposide-treated islets was higher (1.58-fold) than in the DMSO-treated islets. Similar to the results of siTCF7L2 in our previous study, exposure of islets to si*β*-cat decreased *β*-cell proliferation (1.75-fold) and increased *β*-cell apoptosis (3.1-fold; Figure 3e), and these deleterious effects induced by si*β*-cat could not be reversed by geniposide.

These results suggested that the *β*-catenin/TCF7L2 signals have a critical role in the geniposide-mediated protective effect on *β*-cell survival. Geniposide might counteract the Exen(9–39) disadvantage through upregulating TCF7L2 and GLP-1R expressions, which lead to activation of GLP-1R downstream pathways.

### Geniposide treatment normalized blood glucose levels in db/db and HFD mice accompanied by increased *β*-cell mass

The majority of type 2 diabetes rodent animal models are obese, by either genetic or dietary means. Here we used both HFD mice and db/db mice to evaluate the antidiabetic effect of geniposide. In our preliminary experiment, we tried three different doses ranging from 100 to 300 mg/kg. All three doses showed significant glucose-lowering effects compared with saline vehicle-treated animals (data not shown); therefore, the dose of 100 mg/kg was chosen for subsequent experiments.

**Figure 4:**
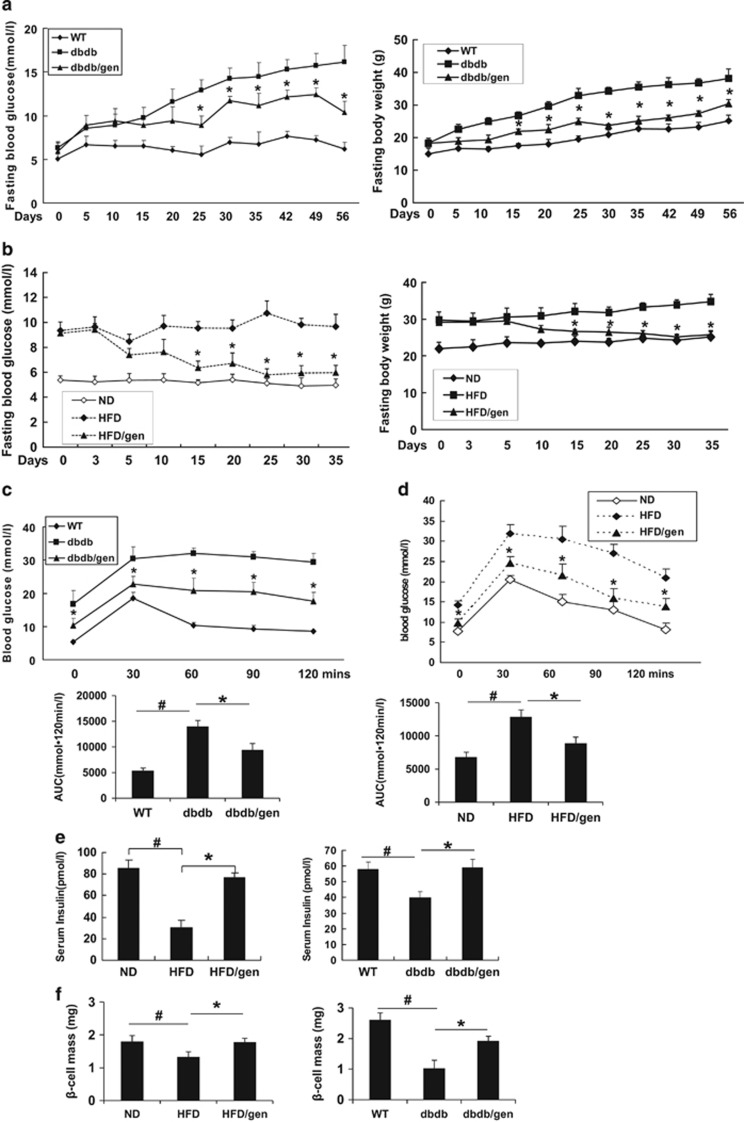
Geniposide normalized blood glucose levels and improved IPGTT in db/db mice and HFD mice. (**a**, **b**) Effects of geniposide on the fasting glucose levels and body weights in db/db mice and HFD mice. (**c**, **d**) Results of IPGTT and AUC. After 5- to 8-week geniposide treatment, IPGTT was performed after 12 h fast with 2 mg/g BW of glucose in control, geniposide- or vehicle-treated mice. (**e**) Serum insulin levels of fasting HFD and db/db mice with/without geniposide treatment. (^#^*P*<0.05 to WT or ND group; *P<0.05 to vehicle group). Data are shown as mean±S.E., *n*=12. (**f**) *β*-cell mass as product of pancreas mass and insulin-positive area divided by section area. Six consecutive sections from each pancreas (nine mice per group) were used for *β*-cell mass measurements. (^#^*P*<0.05 to WT or ND group; *P<0.05 to vehicle group)

To assess the ability of geniposide to prevent progression of diabetes, we initially examined its effect in 4-week prediabetic db/db mice by oral gavage at a dosage of 100 mg/kg daily for 56 days. The vehicle db/db group developed diabetes at 6 weeks of age, and fasting blood glucose levels continued to increase over time. However, prediabetic db/db mice treated with geniposide maintained normal glucose levels until 8 weeks of age and maintained lower glucose levels on subsequent days compared with vehicle-treated db/db mice (Figure 4a).

To further confirm the *in vivo* effect of geniposide, another widely used obese T2DM mouse model, 12-week HFD-induced diabetic mice was administered geniposide for 35 days. The 12-week HFD mice showed a marked increase in fasting blood glucose levels compared with the levels in normal-diet (ND) mice (Figure 4b). Geniposide exhibited a hypoglycemic effect on HFD mice after 15 days of treatment compared with vehicle-treated HFD mice, and this effect continued until the end of the experiment.

In parallel, the response to intraperitoneal glucose challenge (IPGTT) was impaired both in db/db mice and HFD mice, which resulted in significant increases of glucose levels after glucose injection (Figures 4c and d). Geniposide administration protected the diabetic mice from such increases, and lowered blood glucose levels at all time points during the IPGTT.

**Figure 5:**
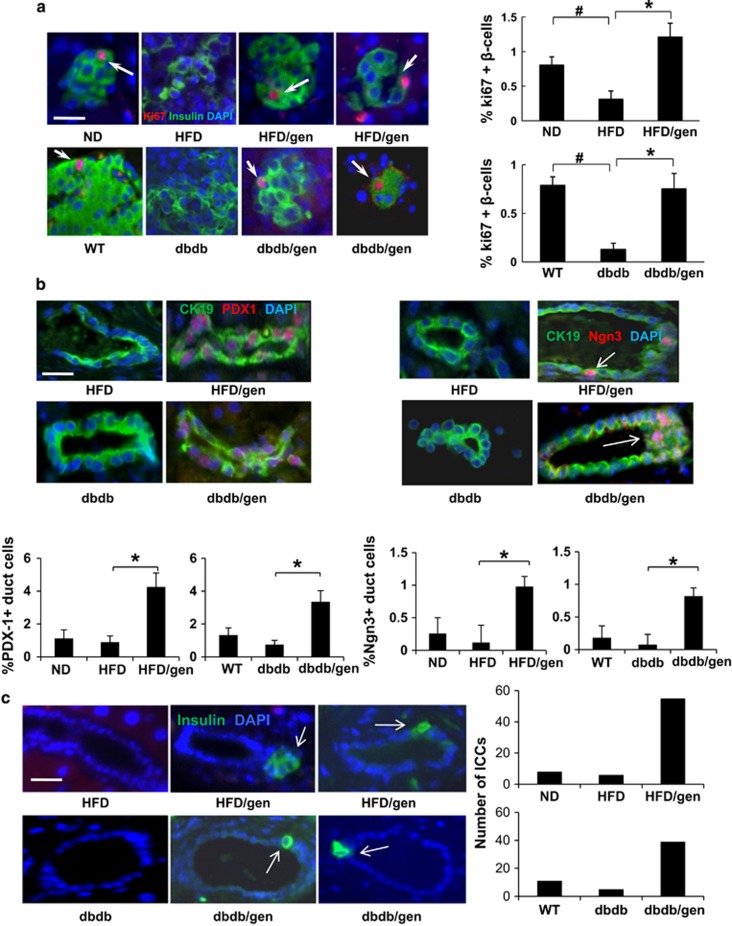
Geniposide promoted *β*-cell regeneration *in vivo*. (**a**) Proliferation of *β*-cell was measured in mice pancreatic sections by triple staining for Ki67 in red (indicated by white arrows), insulin in green, and DAPI in blue. (**b**) The PDX-1 and Ngn3 expressions in CK19-positive ductal epithelial cells. Representative images of ductal epithelial cells after triple staining for PDX-1 or Ngn3 in red, CK19 in green, and DAPI in blue were shown. (**c**) Small ICCs originated in the vicinity of the ducts. Representative pictures of ICCs were shown as insulin-positive staining (indicated by white arrows). Values are representative of four slides spanning the whole pancreas of each mouse and six mice per group. Data are shown as mean±S.E. ( ^#^*P*<0.01 vehicle to WT or ND group; *P<0.01 geniposide to vehicle group). Scale bars, 20 *μ*m

Various reagents that increase plasma insulin levels and exert hypoglycemic effects in db/db mice have been reported.^27, 28, 29^ Here we noticed that geniposide significantly elevated insulin levels in diabetic mice compared with the levels in vehicle-treated diabetic mice (2.2-fold and 1.6-fold higher than corresponding vehicle-treated controls in HFD and db/db mice, respectively; Figure 4e). Immunostaining for *β*-cells showed reduced *β*-cell mass (Figure 4f) and deteriorated islet morphology (Figure 5a) in the vehicle-treated HFD and db/db mice. In contrast, the geniposide-treated group exhibited normal islet morphology (Figure 5a) and increased *β*-cell mass (a 1.4-fold increase in HFD mice and a 1.8-fold increase in db/db mice compared to the *β*-cell mass in corresponding vehicle-treated mice; Figure 4f), which can partially account for the increased insulin levels.

In addition, the body weight gain of geniposide-treated db/db and HFD mice was lower than that of the corresponding vehicle-treated control mice (Figures 4a and b). However, food intake was not significantly impacted by geniposide treatment (Supplementary Figure S1). Both HFD and db/db mice are obese diabetic animal models; therefore, we measured serum total cholesterol and triglyceride content (Supplementary Figure S2). Geniposide treatment significantly decreased total cholesterol levels, but had no remarkable effect on triglyceride levels. The levels of important adipokines, including adiponectin and leptin, were also measured (Supplementary Figure S2). Geniposide treatment reversed the reduction in serum adiponectin levels observed in HFD and db/db mice and suppressed the increased leptin levels observed in HFD mice. These data demonstrate the beneficial metabolic effects of geniposide in HFD and db/db mice.

### Geniposide promoted *β*-cell regeneration *in vivo*

The increase in *β*-cell mass induced by geniposide raised the possibility that this compound may stimulate new *β*-cell formation. Theoretically, new *β*-cells can arise either from differentiation of endocrine progenitor cells or by replication of existing *β*-cells.^4, 30^ In this study, we first detected *β*-cell proliferation by Ki67/insulin immunostaining in pancreatic sections from HFD and db/db mice (Figure 5a). Positive Ki67 staining of *β*-cells was observed in pancreatic sections of ND and wild-type (WT) mice, but was rarely detected in HFD and db/db mice. In contrast, positive Ki67 staining was observed in HFD and db/db mice treated with geniposide (3.8-fold and 5.7-fold higher in HFD and db/db mice, respectively), which was consistent with results obtained from cultured islets.

Expression of the transcription factor PDX-1 in the pancreatic ductal epithelium, a marker for new *β*-cell formation, has been demonstrated in rodent models following partial pancreatectomy,^31^ which was observed in our previous study as well.^10^ Here we observed PDX-1/CK19 double-positive ductal cells in geniposide-treated mice. In contrast, ductal PDX-1 expression was almost undetectable in vehicle-treated mice (4.6-fold and 4.7-fold higher in HFD and db/db mice than in vehicle-treated controls, respectively; Figure 5b). During *β*-cell regeneration, neurogenin 3 (Ngn3) is considered an important marker of the endocrine progenitors that contribute to *β*-cell neogenesis.^32^ The effect of geniposide on Ngn3 expression in ductal cells was measured (Figure 5b). Ngn3 is transiently expressed during endocrine differentiation. Here only 0.95 and 0.82% of ductal cells were Ngn3+ in geniposide-treated HFD and db/db mice, respectively. However, Ngn3 expression was 10-fold higher in geniposide-treated mice than in vehicle-treated mice. Small islet-like cell clusters (ICCs) have been reported to originate as a result of the ductal epithelium differentiation.^33^ As presented in Figure 5c, we also observed ICC structures next to ductal cells in geniposide-treated HFD and db/db mice. The number of ICCs near the pancreatic ducts was counted (ND, 8; HFD, 6; HFD/gen, 55; and WT, 11; db/db, 5; db/db/gen, 39; Figure 5c). These data indicated that geniposide could promote *β*-cell regeneration in diabetic mice.

### Geniposide stimulates ductal cell differentiation by upregulating TCF7L2 expression and activating the JAK2/STAT3 pathway

**Figure 6:**
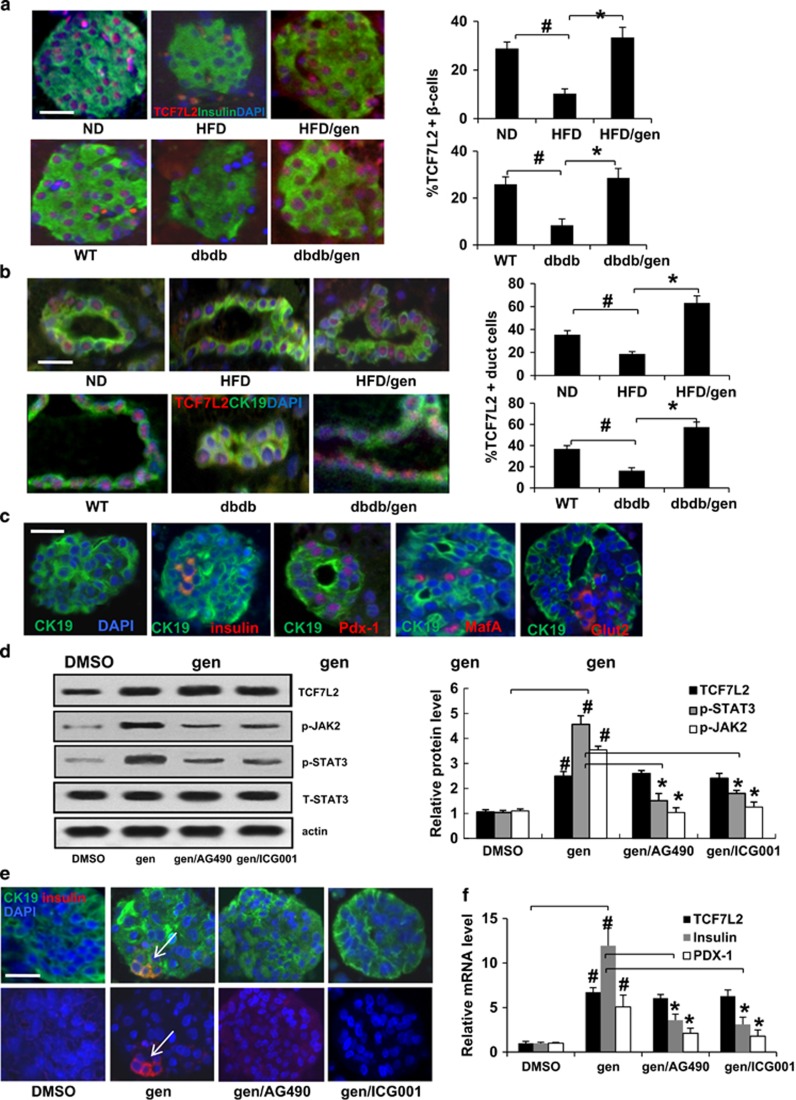
Geniposide triggered ductal epithelial cell to *β*-cell conversion through upregulating TCF7L2 and activating JAK2/STAT3 pathway. TCF7L2 expression in islets *β*-cells (**a**) and in the ductal epithelium cells (**b**) were examined by triple staining of mice pancreatic sections for TCF7L2 in red, insulin, or CK19 in green, and DAPI in blue. Values are representative of three to four slides spanning the whole pancreas of each mouse and six mice per group. Data are shown as mean±S.E. (^#^*P*<0.01 vehicle to WT or ND group; *P<0.01 geniposide to vehicle group). (**c**) Isolated mouse exocrine cells were cultured with 20 *μ*M geniposide for 4 days, then embedded in paraffin and sections were analyzed by immunostaining of CK19, insulin, PDX-1, MafA, and Glut2 to confirm the effect of geniposide on ductal cell differentiation. (**d**) Isolated mouse exocrine cells were cultured with different treatments (20 *μ*M geniposide, 20 mM AG490, and 25 *μ*M ICG001) for 4 days. Upregulation of TCF7L2 and activation of JAK2/STAT3 were analyzed by western blot analysis. Data are shown from three independent experiments. (**e**) Effects of AG490 and ICG001 on the differentiation of ductal epithelial cells. The cells were examined by insulin (red, indicated by white arrows) and CK19 (green), and DAPI (blue) triple staining. (**f**) RT-PCR analyses of Pdx-1, insulin, and TCF7L2 expressions in treated ductal epithelial cells. The experiments were performed in triplicate. The levels of gene expressions were normalized to tubulin (^#^*P*<0.01, geniposide to DMSO-treated group; *P<0.01, geniposide to AG490- or ICG001-treated group). Scale bars, 20 *μ*m

Along with the occurrence of Ki67-positive *β*-cells and PDX-1, Ngn3-positive ductal epithelial cells in geniposide-treated diabetic mice, immunostaining results revealed that TCF7L2 expression (red; Figures 6a and b) in *β*-cells and duct cells was significantly upregulated by geniposide treatment (Figures 6a and b). These observations implied the importance of increased TCF7L2 expression in new *β*-cell formation.

To further confirm the effect of geniposide on ductal cell differentiation, geniposide-treated exocrine cells were embedded in paraffin and sections were analyzed by immunostaining for CK19, insulin, PDX-1, musculoaponeurotic fibrosarcoma oncogene family A (MafA), and glucose transporter 2 (Glut2; Figure 6c). MAF factors are considered to be essential for endocrine differentiation and MAFA acts as a transcriptional factor to cooperate synergistically with NEUROD1 and PDX-1. Glut2 is responsible for glucose uptake in *β*-cells and partially accounts for the glucose-sensing mechanism of *β*-cells. As shown in Figure 6c, the cultured ductal cells formed islet-like clusters as we observed before.^10^ Consistent with the animal experiments, geniposide triggered the differentiation of cultured ductal cells *in vitro* by inducing expression of insulin and PDX-1. Similarly, other proteins expressed in pancreatic progenitors, including MafA and Glut2 were also detected in geniposide-treated ductal cells. A recent publication presented that TCF7L2 could positively regulate expressions of transcription factors like MAFA, PDX-1, and NKX6.1,^34^ further supporting the role of TCF7L2 in new *β*-cell formation as we observed here.

The signals that mediate ductal epithelial cell differentiation are largely unknown. The JAK2/STAT3 pathway has been shown to be involved in *β*-cell neogenesis from acinar cells.^35^ We previously demonstrated that TCF7L2 stimulated ductal cell differentiation through the JAK2/STAT3 pathway.^10^ In this study, mouse exocrine cells were exposed to AG490, a specific JAK2 inhibitor, or ICG001 to clarify whether geniposide-induced ductal epithelial cell differentiation is *β*-catenin/TCF7L2 or JAK2/STAT3 dependent. The western blotting results presented in Figure 6d showed that geniposide treatment markedly stimulated TCF7L2 expression and JAK2/STAT3 activation in exocrine cells, which was strongly inhibited by ICG001. Geniposide treatment induced insulin production within the ductal epithelial cell cluster, which was suppressed by ICG001 and AG490 (Figure 6e). The real-time (RT)-PCR results showed the geniposide treatment increased *PDX-1* and *insulin* mRNA expression in cultured exocrine cells compared with their expression in DMSO-treated cells (Figure 6f). Treatment with ICG001 or AG490 significantly reduced *PDX-1* and *insulin* mRNA expression in geniposide-treated exocrine cells.

## Discussion

Loss of functional *β*-cells is the crucial event in the development of diabetes.^36^ The main goal for diabetes therapy is to prevent loss and dysfunction of existing *β*-cells, meanwhile, to promote new *β*-cell formation. Evidence obtained from both adult humans and animal models has shown that *β*-cell regeneration occurs in a variety of natural and experimental conditions.^37, 38, 39^ Unfortunately, adaptation of *β*-cell mass to insulin demand fails to achieve in T2DM. Novel agents designed to maintain *β*-cell numbers are urgently needed. A recent study reported that FTY720, a potent immunosuppressant isolated from the Chinese herb *Iscaria sinclarii*, is capable of promoting *in vivo β*-cell regeneration in db/db mice.^27^

Natural products are important resources for the antidiabetic drug development.^40^ For instance, metformin is derivative of plant products.^41^ However, the antidiabetic herbs and their bioactive extracts have not been intensively investigated so far. The effects of these compounds on *β*-cell survival and regeneration are still largely unknown.

Here we investigated, for the first time, whether geniposide may trigger new *β*-cell formation both *in vitro* and *in vivo*. In cultured mouse islets, geniposide protected *β*-cells from high glucose- or cytokine-induced apoptosis, and promoted *β*-cell proliferation. We demonstrated that geniposide activated Wnt/*β*-catenin signaling via upregulating TCF7L2 expression. Importantly, geniposide could stimulate *β*-cell replication and induce ICC formation originated from the ductal epithelium both in HFD and db/db mice, with a concomitant increase in TCF7L2 expression. Our findings revealed a novel role for geniposide in the promotion of *β*-cell survival and regeneration through activation of *β*-catenin/TCF7L2 signaling. Interesting, a JBC paper reported that GLP-1 and Exendin 4-activated TCF7L2-dependent Wnt signaling to enhance *β*-cell proliferation,^42^ which provided additional evidence to imply a possible interaction between GLP-1R and Wnt signaling.

Currently, targets of antidiabetic drugs address several separate elements in the *β*-cell that potentially converge on TCF7L2 function.^43^ Direct or indirect stimulation of GLP-1 activity via treatment with DPP-4 inhibitors, GLP-1R agonists, or GPR119 agonists leads to activation of *β*-catenin via increased cAMP levels and improved TCF7L2-driven *β*-cell function.^43^ TCF7L2 could be appreciated as a new target for diabetes treatments. As we described before,^9, 11^ TCF7L2 itself promoted *β*-cell proliferation, protected *β*-cell from apoptosis and improved insulin secretion in cultured islets. In line with our studies, other reports showed mouse islets treated with TCF7L2 siRNA displayed abnormal glucose-stimulated insulin secretion.^12^ In TCF7L2 knockdown rats (generated with specific TCF7L2 morpholino-oligonucleotides), the process of *β*-cell regeneration was significantly inhibited.^8^ Selective deletion of TCF7L2 in mouse pancreas impairs insulin release and glucose homeostasis, indicating the direct role of this factor in controlling *β*-cell function.^13^ Our study implies a correlation between TCF7L2 expression and *β*-cell regeneration. TCF7L2 can trigger differentiation from ductal epithelial cells into *β*-cells *in vitro*.^10^ Similarly, Takamoto *et al.*^14^ reported that in mouse pancreatic *β*-cells, TCF7L2 has a crucial role in glucose homeostasis by regulating *β*-cell mass. DN-TCF7L2 mice (expressing a dominant-negative form of TCF7L2) showed impaired glucose tolerance and decreased insulin secretion. Marked reduction of the *β*-cell area and whole-pancreas insulin content were observed.

Notably, other reports have presented controversial results.^44, 45^ Boj *et al.*^45^ demonstrated that manipulation of islet TCF7L2 expression in adult mice had no significant effects on glucose-stimulated insulin secretion. The authors argued that TCF7L2-related disruption of *β*-cell function is probably an indirect consequence of primary events in liver and elsewhere. Thus, it is still far from a clear understanding of functions of TCF7L2 in metabolism. Novel approaches that address the evident complexity of these systems will be crucial for elucidating the biological functions of TCF7L2.^46^

The underlying mechanisms of *β*-cell regeneration are not fully understood so far. Growth factors have been shown to promote duct cell differentiation and *β*-cell neogenesis. Epidermal growth factor in combination with gastrin,^47^ insulin-like growth factor, transforming growth factor-*β*,^35, 48^ and islet neogenesis-associated protein^49^ have been demonstrated to stimulate *β*-cell growth. The Wnt/*β*-catenin signaling has a crucial role in embryonic development and cell regeneration, differentiation, proliferation, and apoptosis. The JAK2/STAT3 pathway is an important signaling pathway during *β*-cell generation,^35^ and STAT–Wnt interactions were reported before in other cells.^50, 51^ A functional TCF-binding element was detected in the STAT3 promoter, which specifically bound to TCF7L2.^51^ As we described previously, TCF7L2 can trigger differentiation of ductal epithelial cells into *β*-cells *in vitro* by activating the JAK2/STAT3 pathway.^10^ Here we identified that the upregulation of TCF7L2 expression by geniposide *in vitro* could lead to JAK2/STAT3 activation and duct cell differentiation consequently, which further confirmed the involvement of STAT–Wnt interactions in cell differentiation.

On the basis of the crosstalk between GLP-1R signaling and TCF7L2, we used exendin (9–39), and ICG001 to clarify the role of GLP-1R and Wnt signaling in geniposide activity. Interestingly, the regulatory effects of geniposide on p-AKT, p-GSK3*β*, and c-casp3 were strongly blocked by ICG001, whereas exen (9–39) only exhibited mild suppressive effects. Similarly, the protecting effect of geniposide on *β*-cell survival was prevented by ICG001. Moreover, *β*-cell survival could not be preserved by geniposide in the case of *β*-catenin knocked down by si*β*-catenin. Here we noticed that geniposide could enhance the GLP-1R expression. We hypothesize that geniposide could counteract the negative effects of exendin (9–39) by upregulating TCF7L2 and GLP-1R, which can enhance the activation of GLP-1R downstream pathway.

Collectively, we propose a novel mechanism for the effects of geniposide on *β*-cell regeneration and survival. In this model, *β*-catenin/TCF7L2 is the core component required for the functions of geniposide. Geniposide activates *β*-catenin/TCF7L2 transcription complex, which could lead to *β*-cell regeneration via stimulating *β*-cell proliferation and differentiation. Meanwhile, geniposide promoted *β*-cell survival by inhibiting c-casp3 level to suppress *β*-cell apoptosis. Our data support *β*-catenin/TCF7L2 as a possible target for diabetes treatment to promote new *β*-cell formation.

The latest report published in August 2014 by a group at Lund University stated new information that TCF7L2 could positively regulate expressions of transcription factors like MAFA, PDX-1, and NKX6.1, which are crucial for *β*-cell neogenesis, through targeting on insulin gene enhancer-binding protein-1 (ISL1).^34^ This finding provides additional evidence to imply the role of TCF7L2 in *β*-cell regeneration. The association between geniposide and ISL1 needs to be further investigated in our future studies.

## Materials and Methods

### Reagent

Geniposide (purity >98%) was purchased from the National Institute for the Control of Pharmaceutical and Biological Products (Beijing, China). AG490, Exendin (9–39) were from Sigma (St. Louis, MO, USA), and ICG001 was from Selleckchem (Houston, TX, USA).

### Animals

All animal experiments were conducted in accordance with Provisions and General Recommendation of Chinese Experimental Animals Administration Legislation and approved by the Research Animal Care Committee of Nanjing Medical University. The animals were housed in a temperature-controlled room with a 12-h light/dark cycle and were allowed free access to food and water in the course of experiments. Four-week-month old male C57BL/6J mice (SLAC Laboratory Animals, Shanghai, China) were fed with a HFD (60 kcal% fat, D12492, Research Diets, New Brunswick, NJ, USA)^10^ or normal chow diet. Geniposide intervention (100 mg/kg) was initiated after 12 weeks of the HFD. Four-week old male C57Bl/KsJ (BKS) mice and BKS.Cg-Dock7^m^ +/+ Lepr^db^/JNju (db/db) mice were ordered from Model Animal Research Center of Nanjing University. Geniposide solution was prepared in 0.9% NaCl and delivered by oral gavage at dosage of 100 mg/kg daily. The control group was given vehicle. Geniposide or vehicle was given for additional 35–56 days. A total of 12 mice in each group were used.

### Intraperitoneal glucose tolerance tests (IPGTTs)

For IPGTTs, mice were fasted 12 h overnight and injected intraperitoneally with glucose at a dose of 2 mg/g body weight. Blood samples were obtained at time points 0, 30, 60, 90, and 120 min for glucose measurements using a Glucometer (Accu-Chek Active; Roche, Indianapolis, IN, USA). Insulin was determined using a mouse insulin ELISA kit (Alpco, Windham, NH, USA).

### Analysis of *β*-cell mass

*β*-cell mass was measured as previously described.^10^ In brief, pancreatic sections (spanning the width of the pancreas, which was cut along the head–tail axis) were stained with anti-mouse insulin antibody (ab7842, Abcam, Cambridge, MA, USA) and scanned by a Nikon MEA53200 (Nikon, Tokyo, Japan) microscope. The cross-sectional areas of pancreas and *β*-cells were determined by NIS-Elements software (Nikon). *β*-cell mass/pancreas was estimated by the product of the relative cross-sectional area of *β*-cells per total tissue and the weight of the pancreas.

### Mouse pancreatic islets, exocrine cell isolation and culture

Mouse islets were isolated from C57BL/6J mice (SLAC Laboratory Animals) by common bile duct perfusion using Collagenase type 4 (Worthington, Lakewood, NJ, USA) as described previously^11^ and cultured in RPMI 1640 containing 11.1 mmol/l glucose, 100 U/ml penicillin, 100 mg/ml streptomycin, and 10% FCS (Invitrogen, Carlsbad, CA, USA). Isolated pancreatic exocrine cells from islet isolation were cultured in DMEM supplemented with 10% FCS and penicillin–streptomycin (1%).

### Min6 cell culture

Min6 cells were obtained from ATCC (American Type Culture Collection, Manassas, VA, USA) and maintained in 5 mM glucose DMEM, supplemented with 10% FBS (Invitrogen), 50 mmol/l b-mercaptoethanol, 100 U/ml penicillin, and 0.1 mg/ml streptomycin in 5% CO_2_ at 37 °C.

### Treatments of diabetic stimuli

For treatment of islets or Min6 cells with diabetic stimuli, the culture medium contained 33.3 mM glucose or 2 ng/ml recombinant IL-1*β* plus 1000 U/ml recombinant IFN-*γ* (ILIF; R&D Systems) with geniposide (20 *μ*M) for 3 days, cells treated with DMSO served as a control.

### RNA interference transfection

The small interfering RNA transfection experiment was performed by using lipofectamine 2000 reagent (Liptofectamine2000; Invitrogen) according to the manufacturer's instructions. Islets were transfected with 100 nmol/l siRNA against *β*-catenin (sc-29210, Santa Cruz, Dallas, TX, USA) or scramble siRNA (sc-37007, Santa Cruz). After transfection for 24 h, the medium was aspirated and replaced by fresh culture medium with/without geniposide for the next 3-day culture.

### Immunofluorescence staining

Pancreatic tissue and cultured mouse islets were processed as previously described.^9^ Details of antibodies used and the staining procedures were provided in the supplemental files.

### RNA extraction and RT-PCR

Total RNA was isolated from cultured mouse pancreatic islets or exocrine cells as described previously.^10^ For quantitative analysis, Applied Biosystems StepOne Real-Time PCR system (Applied Biosystems, Carlsbad, CA, USA) with a commercial kit (Power SYBR Green PCR Master Mix; Applied Biosystems) was used. Primers used were provided in Supplementary Files.

### Nuclear fractionation

Nuclear and cytoplasm extractions of Min6 cells were performed according to the instructions of NE-PER Nuclear and Cytoplasm Extraction Reagents (Pierce Biotechnology, Rockford, IL, USA). The purity of fractions was analyzed by probing the membranes with anti-GAPDH for cytosolic and anti-PARP for nuclear extracts.

### Western blot analysis

Cultured islets or Min6 cells were washed in PBS and lysed. PVDF membranes were incubated with anti-TCF7L2 (#2565), anti-actin (#4967), anti-p-AKT (Serine473, #9271), anti-AKT (#9272), anti-p-GSK3*β* (Ser9 #9336), anti-PARP (#9542), anti-GAPDH (#2118), anti-c-casp3 (#9661), anti-stat3 (#9132), anti-p-stat3 (Tyr705, #9131), anti-PKA C-α ( #5842; all from Cell Signaling, Danvers, MA, USA), anti-*β*-catenin (ab6302), anti-GLP-1R (ab39072), anti-p-Jak2 (ab68268; all from Abcam), followed by incubation with horseradish-peroxidase-linked IgG peroxidase. The bands were visualized and densities of the bands were analyzed using Tanon ChemImaging Systems (Nanjing, China).

### Statistical analysis

Data are presented as means±S.D. and were analyzed by paired Student's *t*-test or by analysis of variance with a Bonferroni correction for multiple group comparisons.

## Abbreviations

GLP-1R: glucagon-like peptide 1 receptor
GSIS: glucose-stimulated insulin secretion
Glut2: glucose transporter 2
HFD: high-fat diet
ICCs: small islet-like cell clusters
IPGTT: intraperitoneal glucose tolerance test
MafA: musculoaponeurotic fibrosarcoma oncogene family A
ND: normal diet
Ngn3: neurogenin 3
PDX-1: pancreatic and duodenal homeobox 1
TCF7L2: T-cell factor 7-like 2
T2DM: type 2 diabetes
WT: wild type
