# Supplementary material for: Geniposide promotes beta-cell regeneration and survival through regulating β-catenin/TCF7L2 pathway
Source: Cell Death Dis. 2015 May 7;6(5):e1746–. doi: 10.1038/cddis.2015.107 (PMC4669687; doi:10.1038/cddis.2015.107)
Supplement: Supplementary Information [file cddis2015107x1.doc]

**Supplementary information**

**Immunohistochemistry.** The 4-μm paraffin sections of mouse pancreases were deparaffinized, rehydrated, antigen unmasking and incubated overnight at 4ºC with anti- insulin (ab7842, Abcam), anti-Ki67 (550609, BD Pharmingen), anti–TCF7L2 (Cell Signaling), anti-CK19 (ab15463), anti-PDX-1 (ab47267), anti-MafA (ab17976), anti-Ngn3 (ab38548), anti-Glut2 (ab104622) antibodies (all from Abcam) followed by fluorescein– or Cy3-conjugated secondary antibodies (Jackson ImmunoResearch Laboratories). Slides were mounted with Vectashield with 4’6-diamidino-2-phenylindole (DAPI) (Vector Labs). Cultured mouse islets were ﬁxed with 4% paraformaldehyde followed by permeabilization with 0.5% Triton X-100. Cell proliferation was measured by staining with anti-Ki67 (BD Pharmingen), followed by detection with Cy3-conjugated secondary antibody (Jackson ImmunoResearch Laboratories). Apoptosis was analyzed by the terminal deoxynucleotidyl transferase-mediated dUTP nick-end labeling (TUNEL) technique according to the manufacturer’s instructions (In Situ Cell Death Detection Kit, TMR red; Roche Diagnostics). Islets were double/triple stained with anti-insulin antibody (ab7842, Abcam), followed by detection using fluorescein-conjugated secondary antibody (Jackson) and embedded in Vectashield mounting medium (Vector Labs). Fluorescence was analyzed using an Nikon MEA53200 (Nikon) microscope.

**Glucose-stimulated insulin secretion (GSIS).** For acute insulin release, after 3-day treatment islets were washed and pre-incubated (30 min) in Krebs-Ringer bicarbonate buffer (KRB) containing 2.8 mM glucose. The KRB was then replaced by KRB containing 2.8 mM glucose for 1 h (basal), followed by additional 1 h incubation in KRB containing 16.7 mM glucose (stimulated). For insulin content, cells were extracted with 0.18 N HCl in 70% ethanol. Insulin was determined using a mouse insulin ELISA kit (Alpco, Windham, NH).

**Mouse primers** used were:

insulin: 5-TTCTTCTACACACCCA-3

5-CTAGTTGCAGTAGTTCT-3;

tubulin: 5-GTTGGCCAGGCTGGTGTCCAG-3

5-CTGTGATGAGCTGCTCAGGGTGG-3 ;

IL-1β: 5-GACCTTCCAGGATGAGGACA-3

5-AGCTCATATGGGTCCGACAG-3 ;

PDX-1: 5-GAGGACCCGTACTGCCTACA-3

5-CGGGGTCCCGCTACTACGTT-3

TCF7L2: 5-CAGGGAAGAACAGGCAAAAT-3

5-GGGGGAGGCGAGTCTAGTAA-3;

CyclinD1: 5- GGAGATTGTGCCATCCATGC -3

5- TCTTCTTCAAGGGCTCCAGG -3

FigureS1. Effect of geniposide on food intake**.** Data are shown as mean ± SE, n=12. (**#**p<0.05 db/db to WT).

FigureS2. **(A)** Beneficial effects of geniposide on serum cholesterol and triglyceride (TG) levels in HFD and *db/db* mice. **(B)** Effects of geniposide on leptin and adiponectin levels in *db/db* and HFD mice. ( **#** p<0.05 to WT or ND. * p<0.05 to vehicle)

FigureS3. Effect of geniposide on insulin secretion. Mouse islets were exposed to diabetic stimuli with geniposide (20μM) or DMSO as control. GSIS assay was performed after 3-day culture. Basal or stimulated insulin secretion indicated the amount secreted during 1-h incubations at 2.8 or 16.7 mM glucose respectively, normalized to whole insulin content. (* p<0.05, geniposde to DMSO). Data are shown as mean ± SE from three independent experiments.
